# Variability in the Branching Pattern of the Internal Iliac Artery in Indian Population and Its Clinical Importance

**DOI:** 10.1155/2014/597103

**Published:** 2014-12-15

**Authors:** Sumathilatha Sakthivelavan, Sharmila Aristotle, Anandarani Sivanandan, Sakthivelavan Sendiladibban, Christilda Felicia Jebakani

**Affiliations:** ^1^Department of Anatomy, Madha Medical College and Hospital, Chennai, Tamil Nadu 600122, India; ^2^Department of Anatomy, SRM Medical College and Hospital, Chennai 603203, India; ^3^Department of Anatomy, Sri Ramachandra Medical College and Hospital, Chennai 600116, India; ^4^Department of Physiology, Madha Dental College and Hospital, Chennai 600122, India

## Abstract

Internal iliac artery (IIA) is one of the terminal branches of the common iliac artery and is the prime artery of pelvis. The artery has many parietal and visceral branches and hence the variations are frequently noted. The larger branches, namely, the inferior gluteal artery, the superior gluteal artery, and the internal pudendal artery, show sufficient regularity in their patterns of origin to allow typing. The variability of the IIA and its branching pattern were studied by dissecting sixty-eight male pelvic halves (34 right and 34 left) and forty-eight female pelvic halves (24 right and 24 left sides). In significant number of specimens, IIA terminated without dividing into 2 trunks as against the usual description. There was also considerable interchange of branches between the 2 terminal divisions. The patterns of branching noted were grouped as per Adachi's classification. The incidence was noted to be as follows: type Ia in 60.6%, type Ib in 2.6%, type IIa in 15.8%, and type III in 21%. The other types were not observed in this study. *Conclusion*. Interventions in the pelvic region must take into account the variability of the IIA and its branches that can modify the expected relations and may lead to undesired hemorrhagic or embolic accidents.

## 1. Introduction

Internal iliac artery (IIA) also called as hypogastric artery according to the older terminology is given off by the common iliac artery (CIA) at its bifurcation anterior to the pelvic brim at the level of the sacroiliac joint. The artery descends posteriorly within the pelvic cavity towards the greater sciatic foramen. At the upper border of this foramen, it ends by dividing into anterior and posterior divisions. The visceral branches of the anterior division are superior vesical artery (SV), inferior vesical artery (IV), middle rectal artery (MR), uterine and vaginal arteries. The last two branches are present in females, wherein the vaginal artery replaces the inferior vesical artery. The parietal branches of the anterior division are obturator artery (OB), inferior gluteal artery (IG), and internal pudendal artery (IP). The branches from the posterior division are all parietal, namely, iliolumbar artery (IL), lateral sacral artery (LS), and superior gluteal artery (SG) [1]. IIA has multiple variations in the branching pattern. Adachi [2] classified the patterns into five different types, based on its large parietal branches, namely, SG, IG and IP. Only these branches showed sufficient regularity in their origins to enable grouping into different patterns. He described the following types: type I: the superior gluteal artery arises separately from the IIA, and the inferior gluteal and internal pudendal vessels are given off by a common trunk. If the latter divides within the pelvis it is considered to be type Ia, whereas if the bifurcation occurs below the pelvic floor it is classified as type Ib (62 sides; 51.2%); type II: the superior and inferior gluteal arteries arise by a common trunk and the internal pudendal vessel separately. If the trunk common to the two gluteal arteries divides within the pelvis it is type IIa and if the division occurs outside the pelvis it is classified as type IIb (28 sides; 23.1%); type III: the 3 branches arise separately from IIA (22 sides; 18.2%); type IV: the three arteries arise by a common trunk. The subtyping in this group is based on the sites of origin of the superior gluteal and the internal pudendal arteries from the parent stem. In type IVa the trunk first gives rise to the SG before bifurcating into the other two branches; in type IVb the internal pudendal is the first vessel to spring from the common trunk, which then divides into superior and inferior gluteal arteries (5 sides; 4.1%); type V: the internal pudendal and the superior gluteal arteries arise from a common trunk, and the inferior gluteal has a separate origin (1 side; 0.8%) [2].

The concentration of organs and the anatomical structures within the closely packed confines of the pelvis makes the study of vascular patterns and their variations of much importance. Moreover the role played by embryonic umbilical artery, which later on becomes a regressed constituent of the IIA complex in the development of anomalous branches or variations of vascular anatomy is of considerable importance [3]. Internal iliac ligation is done for various indications including obstetric reasons for postpartum hemorrhage [4]. Inclusion of posterior division into ligature results in gluteal necrosis. The varying lengths of IIA, combined with its variable terminations, may confuse the surgeon in identifying the anterior division for ligation. The variable origins of the branches from different regions of the IIA, if unnoticed, may result in improper therapeutic embolisation.

This study aims at providing a detailed account of the length of IIA, level and mode of termination of IIA, and branches from the main stem of IIA before its bifurcation and the branching patterns in Indian population are compared with Adachi's classification.

## 2. Materials and Methods 

The study was performed on 116 pelvic halves from 58 embalmed cadavers in the age group of 30–80 years belonging to the Indian population. The specimens included sixty eight male pelvic halves (34 right and 34 left sides) and forty eight female pelvic halves (24 right and 24 left sides). All these cadavers were free of surgical scar or injury in pelvic or gluteal region. The whole pelves were separated at the level of L4-L5 articulation and bisected longitudinally in the midline. The CIA was traced to find the external iliac artery (EIA) extending in line with CIA towards the inguinal ligament and internal iliac artery directed towards the pelvic cavity. The level of bifurcation of CIA into EIA and IIA was noted.

The tributaries of internal iliac vein along with the main trunk were removed in toto, taking care not to injure the branches of IIA. With careful dissection, IIA was traced to its termination into two divisions or branches. The length of IIA from its origin to the termination was measured.

From each division, both parietal and visceral branches were traced. The parietal branches were traced till their exit from the pelvic cavity. The visceral branches were traced till they reach the organ of their destination.

## 3. Results 

### 3.1. Types of Branching

**Figure 1:**
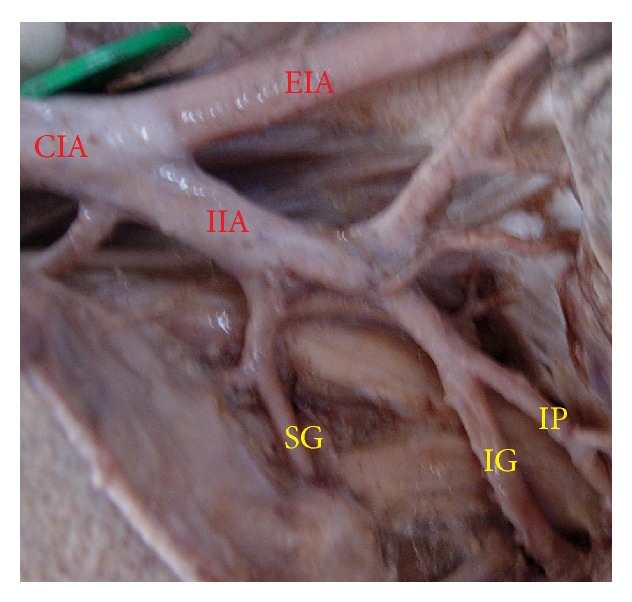
Left pelvic half shows type Ia Adachi's classification—superior gluteal artery arises separately from internal iliac artery and a common trunk for inferior gluteal artery and internal pudendal artery divides proximal to the pelvic floor. IIA: internal iliac artery; IG: inferior gluteal artery; SG: superior gluteal artery; IP: internal pudendal artery.

**Figure 2:**
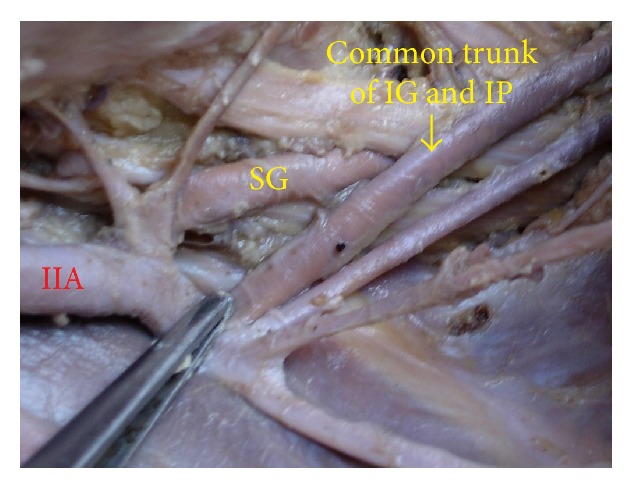
Left pelvic half shows type Ib Adachi's classification—superior gluteal artery arises separately from internal iliac artery and a common trunk for inferior gluteal artery and internal pudendal artery divides distal to the pelvic floor. IIA: internal iliac artery; IG: inferior gluteal artery; SG: superior gluteal artery; IP: internal pudendal artery.

**Figure 3:**
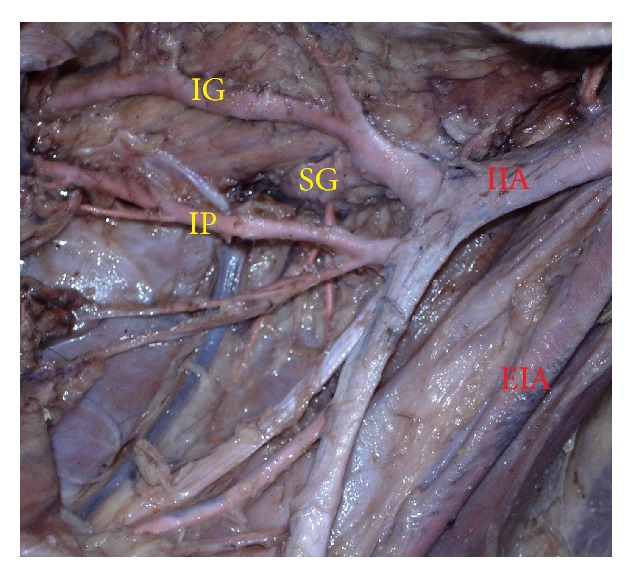
Left pelvic half shows type IIa Adachi's classification—internal pudendal artery arises separately from internal iliac artery while inferior gluteal artery and superior gluteal artery arise by a common trunk which divides proximal to the pelvic floor. IIA: internal iliac artery; EIA: external iliac artery; IG: inferior gluteal artery; SG: superior gluteal artery; IP: internal pudendal artery.

**Figure 4:**
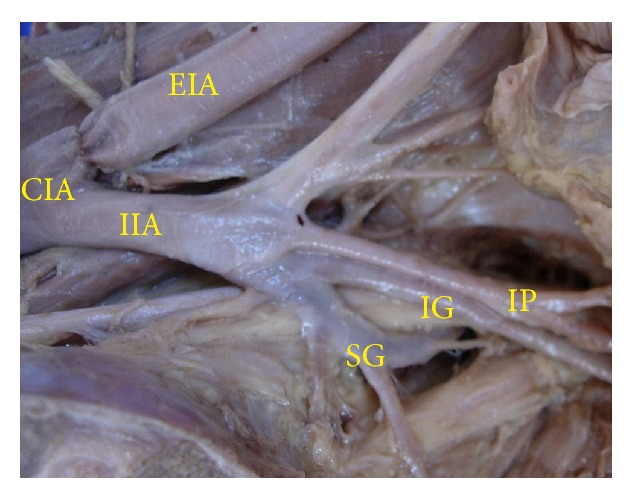
Left pelvic half shows type III Adachi's classification—the three branches, namely, inferior gluteal, superior gluteal, and internal pudendal artery, arise separately from the internal iliac artery. CIA: common iliac artery; IIA: internal iliac artery; EIA: external Iliac artery; IG: inferior gluteal artery; SG: superior gluteal artery; IP: internal pudendal artery.

The superior gluteal artery arose separately from the IIA, and the inferior gluteal and internal pudendal vessels are given off by a common trunk in 63.2%. The common trunk divided within the pelvis, that is, type Ia (Figure 1), in 60.6% (70 specimens; 42 male and 28 female pelvic halves) whereas the bifurcation occurred below the pelvic floor that is, type Ib (Figure 2) in 2.6% (3 specimens; 2 male and 1 female pelvic halves). The superior and inferior gluteal arteries arose by a common trunk that divided proximal to the pelvic floor and the internal pudendal vessel separately that is, type IIa (Figure 3), in 15.8% (18 specimens; 9 male and 9 female pelvic halves). The three branches arose separately from IIA, that is, type III (Figure 4), in 21% (25 specimens; 15 male and 10 female pelvic halves). Types IIb, IV, and V were not noted in any of the specimens.

### 3.2. Extent of the IIA

**Figure 5:**
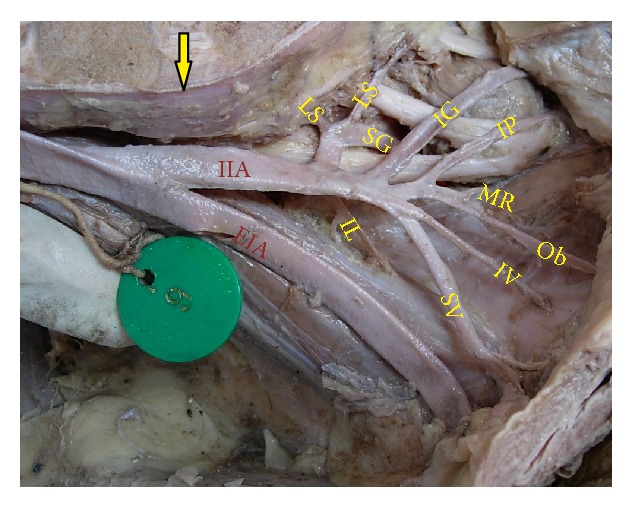
Right half of pelvis showing the bifurcation of IIA at the level of lumbosacral articulation. Arrow: lumbosacral joint; IIA: internal iliac artery; EIA: external iliac artery; IG: inferior gluteal artery; SG: superior gluteal artery; IP: internal pudendal artery; SV: superior vesical artery; IV: inferior vesical artery; MR: middle rectal artery; Ob: obturator artery; LS: lateral sacral artery; IL: Iliolumbar artery.

**Figure 6:**
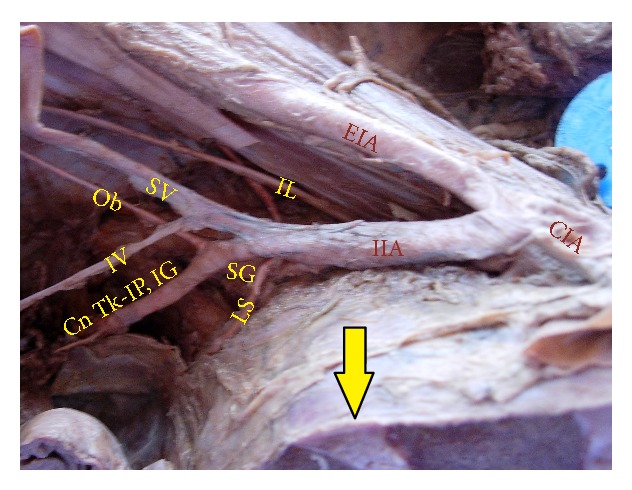
Right half of pelvis showing the bifurcation of IIA above the level of lumbosacral articulation. Arrow: lumbosacral joint; IIA: internal iliac artery; EIA: external iliac artery; CIA: common iliac artery; SG: superior gluteal artery; SV: superior vesical artery; IV: inferior vesical artery; Ob: obturator artery; LS: lateral sacral artery; IL: iliolumbar artery; Cn Tk-IP, IG: Common trunk of internal pudendal and inferior gluteal artery.

The origin of IIA was found to be at the level of lumbosacral articulation (Figure 5) in 94 specimens (81%) and it was above that level (Figure 6) in 22 specimens (19%). The termination of IIA into its divisions or branches was found to be at the level of upper border of greater sciatic notch in 76 specimens (65.5%) while it was at a highly variable position between lumbosacral articulation and greater sciatic notch in 40 specimens (34.5%).

### 3.3. Length of the IIA

**Figure 7:**
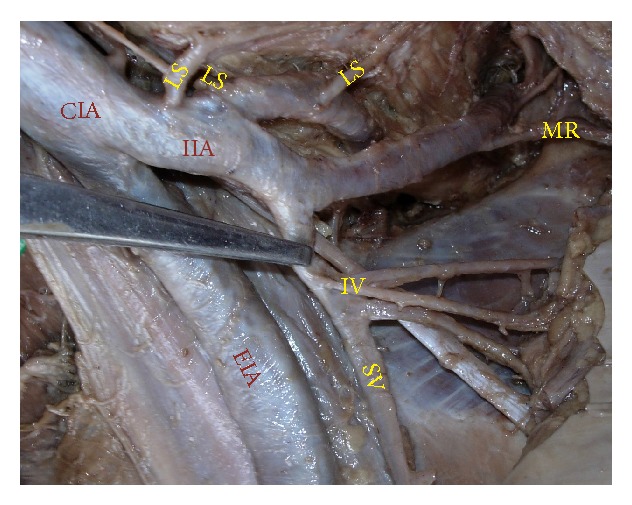
Right half of pelvis showing short internal iliac artery. IIA: internal iliac artery; EIA: external Iliac artery; CIA: common iliac artery; SV: superior vesical artery; IV: inferior vesical artery; MR: middle rectal artery.

**Figure 8:**
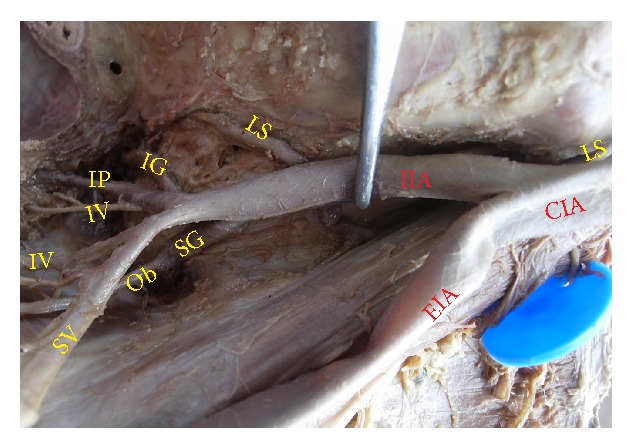
Right half of pelvis showing long internal iliac artery. IIA: internal iliac artery; EIA: external iliac artery; CIA: common iliac artery; SV: superior vesical artery; IV: inferior vesical artery; IG: inferior gluteal artery; SG: superior gluteal artery; IP: internal pudendal artery.

The length of IIA ranged from the minimum measure of 2.3 cm (Figure 7) to the maximum of 7.1 cm (Figure 8). However, in majority of specimens (67%) the length of IIA ranged from 2.5 to 4.2 cm. The mean length was found to be 3.7 cm.

### 3.4. Termination of the IIA

**Figure 9:**
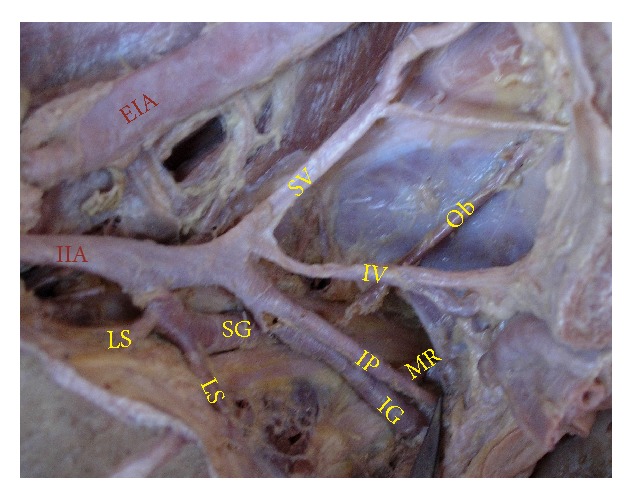
Left pelvic half showing the termination of IIA into anterior and posterior divisions. IIA: internal iliac artery; EIA: external iliac artery; SV: superior vesical artery; IV: inferior vesical artery; IG: inferior gluteal artery; SG: superior gluteal artery; IP: internal pudendal artery; MR: middle rectal artery; Ob: obturator artery.

**Figure 10:**
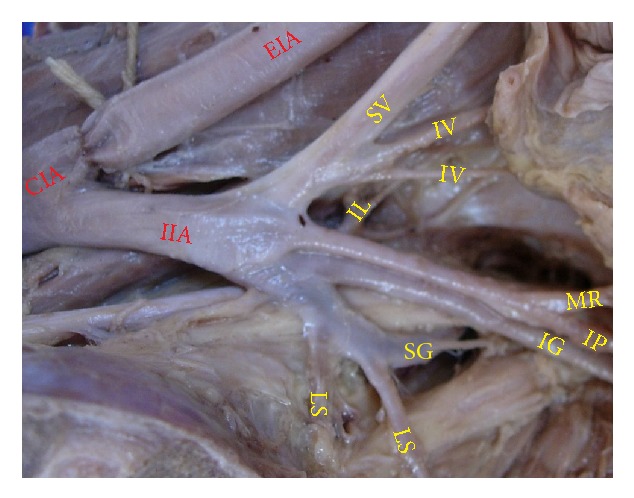
Left pelvic half showing the termination of IIA into spray of branches. CIA: common iliac artery; IIA: internal iliac artery; EIA: external iliac artery; SV: superior vesical artery; IV: inferior vesical artery; LS: lateral sacral artery; IL: iliolumbar artery; IG: inferior gluteal artery; SG: superior gluteal artery; IP: internal pudendal artery; MR: middle rectal artery.

The IIA terminated by dividing into anterior and posterior trunks (Figure 9) in 92 specimens (79.3%) and in the remaining (20.7%) it terminated by giving rise to its principal branches directly without dividing into two trunks (Figure 10).

### 3.5. Branches Arising from Main Stem of the IIA

The main vessel usually does not give rise to any significant named branches, while all of them arise from its divisions. But in this study, one or more branches were found to arise from the main stem of the IIA before its termination in 56.9% of the specimens as shown in Table 1. The branches were IL, LS, and MR in varying proportion of specimens.

### 3.6. Altered Origin of Branches from the Divisions

Some of the branches of the IIA were noted to deviate from their usual origins of the anterior or posterior divisions. IG, OB, and MR which are normally branches of the anterior division, were noted to arise from posterior division in 19.8%, in 6.8%, and in 6.8%, respectively. Lateral sacral artery, usually a branch of posterior division, was found to arise from anterior division in 32.7%.

## 4. Discussion 

The classification of our findings based on Adachi's types is mentioned in Table 2. Type Ia was the most common finding. The frequency of occurrence of type II was less than that of type III. This was against the findings of Adachi and many other studies [5–7] as shown in Table 3. Types IV and V were not found in any of the specimens. On comparison, it is evident that the present findings are in accord with that of the previous studies in type Ia being the most common. No specimen of type IIb, IV, or V is met with in the present study. The other studies that are shown in Table 3 did not reveal type V where a common trunk divides into internal pudendal artery and superior gluteal artery. Types I, II, and III are in the order of I > III > II in the present study as against I > II > III by Adachi's classification. These differences are probably due to variability in the vasculature of different ethnic groups.

The length of IIA ranges from 3 to 4 cm [1]. The longest IIA had been described to be 7.5 cm [8]. The clinical importance underlying the measurement of length of IIA is that, the application of a ligature to IIA may be needed in cases of aneurysms or hemorrhage affecting one of its branches. The degree of facility of applying a ligature to this vessel will mainly depend on its length. If the vessel is short, then it is deeply seated in the pelvis. On the contrary, if artery is longer it is found partly above the cavity. If artery is short, it would be preferable to apply a ligature to the CIA or upon EIA and IIA at their origin [9].

The ligation of the IIA, 5 cm distal to the common iliac bifurcation is said to spare posterior division branches in the vast majority of cases with uterine hemorrhage [10]. This rule may not apply in the presence of IIA, longer than 5 cm as noted in 25.9% in our study.

The branches which arise from anterior and posterior trunks may vary considerably [11]. This fact is rightly supported by the interchange of origins of IG, OB, MR, and LS from the two divisions of IIA. The origin of OB from the posterior division was observed in 0.5% by Kumar and Rath, while in our study it was noted in 6.8%. This origin is advantageous in the cases of surgical ligation or pathological obstruction of the anterior division of internal iliac artery, since there will be sparing of OB and its branches, especially the branch to the head of the femur. The parietal branches of OB are important collaterals in aortoiliac and femoral arterial occlusive diseases. Therefore this may be considered for a possible bypass grafting in cases of ischemic necrosis of head of femur following decreased blood flow through OB, connecting the posterior division to the distal end of the obstruction. Moreover, the increased length of the left OB, owing to the origin from the posterior division of internal iliac, may have an additional advantage while grafting. Thus to avoid complications during surgery, the radiologists and pelvic surgeons should be aware of this rare variation [12].

Embryologically, the umbilical artery has a primary origin from ventral part of aorta and later on, has a secondary origin from the lateral part of aorta and the primary origin disappears. After birth, the greater part of intraabdominal portion of umbilical artery atrophies and is converted into medial umbilical ligament but from the previous part in the pelvis springs the superior vesical artery. The primary axial artery of lower limb is inferior gluteal artery, which springs from secondary umbilical artery [13].

Developmentally, IIA consisted of two main trunks: the medial umbilical ligament (superior vesical artery) and inferior gluteal artery. Most of the branches originate from the two trunks and the junction between them [14]. The OB arises comparatively late in development as a supply to a plexus, which in turn is joined by the axial artery of lower limb that accompanies the sciatic nerve [15]. The branches from the main stem of IIA were noted in majority of the specimens (56.9%) in contrast to the description by standard textbooks [1]. Among these IL and LS showed higher incidence of 16.4% and 19.8%, respectively. Origin of IL from the main stem of IIA was described as “Level C” and it was noted in 52.5% in a study. Surgical interventions in the lumbar, sacral, and pelvic regions must take into account the variable origins of the IL from the iliac system that can modify the expected topographical relations and may lead to undesired hemorrhagic accidents [16].

Yamaki et al. proposed a new classification in which the umbilical artery was excluded from the classification of the mode of branching of this artery. The superior gluteal, inferior gluteal, and internal pudendal arteries were determined to be the principal branches of the IIA and the mode of branching of this artery were classified into 4 groups [17].

Understanding IIA anatomy is essential to minimize intraoperative blood loss and other complications [10]. Internal iliac angiogram is done for various indications. To avoid confusion due to anatomical variations, the anterior and posterior divisions are referred to as superior and inferior trunks radiologically [4]. Although rare, pelvic embolisation may lead to complications like foot drop and sciatic pain. In order to prevent these complications, SG is protected by placing the coil at its origin before performing particle embolisation of IG [18]. The radiologist performing such procedure should be aware of variations in the branching of IIA prior to the interventions.

## 5. Conclusion

The study reveals the variations in IIA anatomy as against the standard depiction of the artery. In 20.7% of specimens, IIA gave origin to larger terminal branches without dividing into two trunks. In most of the specimens one or two branches arose from the main stem of IIA even before its bifurcation. Interchange of the origin of some branches between the two trunks was observed. The highest proportion of cases displayed the branching patterns, namely, type Ia of Adachi's classification. The order of frequency in Indian population was I > III > II as against most other studies. Understanding IIA anatomy is essential to minimize intraoperative blood loss and other complications.

## Figures and Tables

**Table 1:** Branches originating from the main stem of internal iliac artery.

Branch	Number	Percentage
Iliolumbar artery	19	16.4
Lateral sacral artery	23	19.8
Iliolumbar artery and lateral sacral artery as separate branches	4	3.4
Iliolumbar artery and lateral sacral artery as a common trunk	8	6.9
Iliolumbar artery as separate branch and lateral sacral artery and middle rectal artery as common trunk	4	3.4
Common trunk for iliolumbar artery and middle rectal artery	4	3.4
Lateral sacral artery and middle rectal artery as separate branches	4	3.4
No branch before division	50	43.1
Total	**116**	**100**

**Table 2:** Findings based on Adachi's classification.

Types	Male (68)		Female (48)		Total (116)	
	Number	Percentage	Number	Percentage	Number	Percentage
Ia	42	61.8	28	58.3	70	60.6
Ib	2	2.9	1	2	3	2.6
IIa	9	13.2	9	18.8	18	15.8
IIb						
III	15	22.1	10	20.8	25	21

**Table 3:** Studies based on Adachi's classification.

Study	Ia	Ib	IIa	IIb	III	IVa	IVb	V
Adachi (1928) [2] (118 specimens) %	51.2		23.1		18.2	4.1		0.8

Braithwaite (1952) [5] (169 specimens) %	48.5	10	11.8	3.5	22.5	2.4	1.2	—

Fischer (1959) [6] (50 specimens) %	46	4	12	14	16	2	6	—

Roberts and Krishingner (1967) [7] (167 specimens) %	45	6	25	1.8	14.4	5.4	1.8	—

Our study (116 specimens) %	60.6	2.6	15.8	—	21		—	—
